# The taTME learning curve for mid-low rectal cancer: a single-center experience in China

**DOI:** 10.1186/s12957-022-02763-3

**Published:** 2022-09-23

**Authors:** Fengming Xu, Yiqiao Zhang, Jiafu Yan, Bowen Xu, Guocong Wu, Zhengyang Yang, Liting Sun, Xiao Zhang, Hongwei Yao, Zhongtao Zhang

**Affiliations:** 1 Department of General Surgery, Beijing Friendship Hospital, Capital Medical University, 95 Yong-an Rd, Xi-Cheng District, Beijing, 100050 China; 2 National Clinical Research Center for Digestive Diseases, 95 Yong-an Rd, Xi-Cheng District, 100050 Beijing, China; 3 Department of Cardiovascular Medicine, Beijing Anzhen Hospital, Capital Medical University, 2 An-zhen Rd, Chao-Yang District, Beijing, 100029 China

**Keywords:** Transanal total mesorectal excision, Learning curve, CUSUM, Rectal neoplasms

## Abstract

**Purpose:**

As transanal total mesorectal excision (taTME) is performed worldwide, the optimization of existing training and guidance programs to enhance new taTME learners’ competence in performing this procedure is warranted. This study aimed to evaluate the taTME learning curve in patients with mid-low rectal cancer.

**Methods:**

Patients who underwent taTME for mid-low rectal cancer between October 2015 and August 2021 at a single center were included. A cumulative sum (CUSUM) learning curve analysis was performed with the total operation time as the study outcome. The learning curve was analyzed using risk-adjusted CUSUM analysis, with postoperative complications and anastomotic leakage (AL) as outcomes.

**Results:**

In total, 104 consecutive patients were included in this study. The CUSUM learning curve for total operative time started declining after 42 cases (309.1 ± 84.4 vs. 220.2 ± 46.4, *P* < 0.001). The risk-adjusted CUSUM (RA-CUSUM) learning curve for postoperative complications fluctuated in cases 44–75 and declined significantly after case 75. The RA-CUSUM learning curve for AL declined after 68 cases.

**Conclusions:**

taTME had learning curves of 42, 75, and 68 cases for total operative time, postoperative complications, and AL, respectively. A surgeon may require 42 and 75 cases to achieve “proficiency” and “mastery” in taTME procedures, respectively.

**Supplementary Information:**

The online version contains supplementary material available at 10.1186/s12957-022-02763-3.

## Introduction

In 2010, Sylla et al. [1] first proposed transanal TME (taTME) to improve the quality of surgical specimens for middle and low rectal cancers. Since its introduction to surgical practice, taTME has been conceived as “a new solution to some old problems [2].” In particular, taTME potentially achieves circumferential resection margin (CRM) and distal resection margin (DRM) in male patients with obesity and narrow pelvis, and it has been gradually accepted by some colorectal surgeons as a safe and feasible procedure [3, 4]. However, high multifocal pattern local recurrence rates were reported in two studies on taTME oncological outcomes [5, 6].

As the taTME procedure is widely performed worldwide, evidence regarding the reliability of this technique in terms of oncology safety is increasing [7, 8]. This appears to encourage more colorectal surgeons to attempt this procedure. Although taTME has unique advantages in the treatment of low and middle rectal cancer over relatively complex surgeries, such as laparoscopic radical rectal cancer, it often has an inherent learning curve [9]. In a recent study in the Netherlands that reported local recurrence rates during taTME implementation in a structured national training program, the authors noted that even when surgeons were supervised, there was a significant learning-curve effect in the first 10 cases [6].

Therefore, it is crucial to study the taTME learning curve to determine the learning stage of the technique. This study aimed to evaluate the learning curve for taTME to determine the number of procedures required to achieve proficiency and mastery, respectively.

## Methods

### Patient selection

One-hundred and four consecutive patients who underwent taTME for mid-low rectal cancer between October 2015 and August 2021 at a single center (Beijing Friendship Hospital, Capital Medical University, China) were included. Patient data were included in an institutional database of prospective records and retrospectively analyzed for the purposes of this study. Inclusion criteria included adults with a diagnosis of rectal adenocarcinoma located up to 10 cm from the anal verge undergoing elective taTME and the absence of exclusion criteria. Exclusion criteria were specific contraindications to laparoscopy, procedures performed in an emergency setting (intestinal obstruction, perforation, etc.), and histology other than adenocarcinoma. Patients treated through local excision were excluded. All patients underwent preoperative colonoscopy and tumor biopsy. Chest/abdominal computed tomography (CT) and pelvic magnetic resonance imaging (MRI) were performed on all patients, except those with contraindications, after histological diagnosis of rectal cancer to assess clinical staging. This study was approved by the Institutional Research and Ethics Committee.

### Data collection

Collected data included sex, age, BMI, comorbidity (hypertension, diabetes, cardiovascular disease, and respiratory disease), previous abdominal surgery, American Society of Anesthesiologists (ASA) score, preoperative tumor staging, preoperative neoadjuvant therapy, and the distance of the tumor edge from the anal edge (measured using MRI or colonoscopy/CT in patients who could not undergo MRI). Intraoperative variables included total operation time (transabdominal + transanal), type of colorectal reconstruction, creation of a diverting ileostomy, intraoperative blood loss, anal drainage, and conversion to open surgery. The pathological characteristics included specimen quality, tumor dimension, pathological stage, and CRM and DRM statuses. Postoperative outcomes included length of postoperative stay and 30-day postoperative complications (all postoperative complications, reoperation, readmission, and mortality). Local recurrence and survival status were included in the follow-up data.

TME-specimen quality was categorized based on the principles described by Nagtegaal et al. [10]. Specimen quality was assessed jointly by the pathologist and surgeon. Positive CRM was defined as the presence of a tumor or malignant lymph node ≤ 1 mm from the CRM. Local recurrence was defined as radiologic or endoscopic evidence of one or more new pelvic lesions documented during surveillance after removal of the primary tumor, and distant recurrence was defined as disease recurrence outside the pelvis that was not present at the time of diagnosis or surgery.

### Surgical technique

All surgical procedures were performed by the same surgical team. The taTME procedures were performed by 3 laparoscopic colorectal surgeons. Transanal procedures were performed by the same surgeon, and transabdominal procedures were performed by the assisting team; after 14 cases, the transabdominal operation was performed by another member of the team. In the first 14 cases, transabdominal and transanal operations were performed sequentially, and from the 15th case, transabdominal and transanal operations were performed simultaneously. These surgeons had extensive experience in laparoscopic colorectal surgery, and each had performed > 300 laparoscopic rectal resections. All patients underwent preoperative mechanical intestinal preparation 1 day before surgery and prophylactic intravenous infusion of antibiotics 30–60 min before surgery.

#### Transabdominal phase

Laparoscopic exploration, dissection of the inferior mesenteric artery or superior rectal artery root, and mesenteric-membrane dissection were the same as in conventional laparoscopic-assisted TME surgery. Splenic flexure mobilization was performed when necessary for tension-free anastomosis.

#### Transanal phase

After disinfecting and fully expanding the anus, a Lone Star Retractor™ was positioned to better expose the anal canal. Depending on the tumor location, the transanal operation platform and surgical instruments were directly inserted, or intersphincteric resection (ISR) was initially performed transanally.

#### Purse-string suture

Discontinuous annular pre-labeling of the intestinal wall was performed on the mucosal surface of the intestinal wall 1–2 cm from the tumor’s lower edge. A 2–0 suture was used to purse-string suture the muscular layer of the rectal wall at the labeled site and subsequently tighten the purse and tie the knot to close the rectal lumen. The mesorectum was isolated through the anus in the “Holy Plane” of the TME until total mesorectal resection was completed by meeting with the transabdominal operating plane.

According to tumor height and preoperative anorectal function, one of the following two surgical procedures was performed: (1) TME resection and one-stage anastomosis or (2) TME resection with end colostomy. After the anastomosis was completed, it was examined through the anus, and, if necessary, reinforced by manual suture.

In the early stages, surgeons attempted transanal endoscopic microsurgery (TEM), single-incision laparoscopic surgery (SILS) port, and STAR-port platforms (Fig. S1a–c). After 33 cases, the STAR-port platform (Starport, Surgaid Medical, Xiamen, China) was used regularly. The part of the anal canal inserted is made of hard material, which can open the anal canal to better expose the surgical field, and the operation of surgical instruments is more flexible. All patients received postoperative care according to the enhanced recovery after surgery protocol.

### Endpoints

The main outcome of this study was the number of procedures required to achieve proficiency and mastery, respectively. Cumulative summation (CUSUM) analysis was used to evaluate the learning curve, with operative time, incidence of postoperative complications, and anastomotic leakage (AL) as endpoints. The point at which the operative time became consistent with the mean, without further significant changes in terms of operative time, was defined as the point of “proficiency” of the technique [11]. “Mastery” was considered achieved when there was a significant decrease in the learning curve graph with the outcomes of postoperative complications and anastomotic leakage. Operative time was defined as the sum of the transabdominal and transanal operation times. Thirty-day postoperative complications were classified according to the Clavien–Dindo classification [12] to separate minor (I–II) from major complications (III–V). Major surgical postoperative complications were defined as grade 3 or higher complications, excluding extra-abdominal and stoma-related morbidities. AL was diagnosed and graded according to the definition provided by the International Study Group of Rectal Cancer [13].

### Statistical analyses

The CUSUM method is a time-weighted control chart method that has received increasing attention in recent years for detecting the value of learning curves [14]. This method calculates the degree of deviation of each sample observation from the target value and cumulative sum using summation methods. The target time was defined as the mean operative time for all patients. If the CUSUM curve rose, it indicated that the corresponding observation was above the sample mean, and if the curve fell, it indicated that the corresponding observation was below the sample mean. The peak of the curve is the turning point for crossing this surgical learning curve.

Since the CUSUM method is based on purely observing surgical outcomes, this ignores the situation where certain patients with high surgical risk factors have poor surgical performance. The accuracy of the above results was verified using the risk-adjusted CUSUM method (RA-CUSUM) [15], as an extension of the CUSUM method, which could be used to explain the difference between predicted and actual events. It adjusts the risk of preoperative surgical failure for each patient by means of a likelihood-based scoring method to reduce the possibility that those deaths caused by differences in patient mix will not be wrongly attributed to the surgeon. After determining the risk factors in this experiment, the risk probability of each specific value in the data was predicted using risk factor scores, which in turn allowed us to determine the reliability of the CUSUM results.

Postoperative complications and anastomotic leakage were further risk-adjusted CUSUM analyzed. The risk factors associated with outcomes were first identified by univariate logistic regression analysis, and at the same time, by reviewing studies on the risk factors for taTME postoperative complications [16, 17], the risk factors were finally identified: male, BMI ≥ 25, ASA score > 2, tumor height, preoperative neoadjuvant therapy, intraoperative blood loss ≥ 100 ml, and tumor size. These risk factors were included in the multivariate logistic regression analysis to determine the probability of complications (*p*) for each patient, and the independent risk factors were male, ASA score > 2, MRI-N stage. Similarly, the cutoff point of the CUSUM curve was considered as the end of the learning period. Regarding RA-CUSUM with AL, the same method was used for the analysis.

Baseline demographic, clinical, and surgical characteristics as well as crude outcomes of participants are presented as frequencies and percentages for categorical variables, means and standard deviations (SDs), or median and interquartile ranges for continuous variables, depending on whether datasets were normally distributed. Between-group differences in categorical variables were evaluated using the chi-square test. The two-tailed *t*-test (normal distribution) or Mann–Whitney *U*-test (skewed distribution) was used to determine any significant differences between the means or medians of the groups. A normal Q-Q plot was used to assess the normality of the data distribution. All analyses were performed using the Statistical Package for the Social Sciences (version 26.0; IBM, Armonk, NY, USA). Statistical significance was set at *P* < 0.05.

## Results

### Patient characteristics

A total of 104 consecutive patients (77 men, 74.0%) were included in this study. The demographic data, tumor characteristics, and treatment characteristics of the entire cohort are shown in Table 1. The mean age of the cohort was 61.5 years (± *SD* 11.5, range 35–87). The median BMI was 24.2 kg/m^2^ (*IQR* 22.2–26.0). Twenty-one patients (20.2%) underwent abdominal and pelvic surgeries for various reasons. A total of 51 patients (49.0%) received preoperative neoadjuvant chemoradiation therapy. Eighty-four (80.8%) patients were at clinical stage T3–4, 51 (49.0%) had N +, and 22 (21.2%) had positive preoperative MRI-CRM. The median preoperative distance from the lower margin of the tumor to the anal margin was 50.0 mm (*IQR* 40.0–66.8). There were 56 (53.8%) and 48 (46.2%) cases of low rectal and middle rectal cancers, respectively.

**Table 1:** Patient demographics, preoperative staging, and treatment of cancer cases

Variable	Values
Sex, *n* (%)	
Male	77 (74.0)
Female	27 (26.0)
Age, mean ± SD	61.5 ± 11.5
BMI, kg/m^2^, median (IQR)	24.2 (22.2–26.0)
Comorbidity, *n* (%)	
Yes	48 (46.2)
No	56 (53.8)
ASA, *n* (%)	
I	9 (8.7)
II	77 (74.0)
III	18 (17.3)
Neoadjuvant chemoradiation, *n* (%)	
Yes	51 (49.0)
No	53 (51.0)
Previous abdominal surgery, *n* (%)	21 (20.2)
Distance from anal verge, mm, median (IQR)	50.0 (40.0–66.8)
Middle rectum, *n* (%)	48 (46.2)
Lower rectum, *n* (%)	56 (53.8)
Preoperative T stage, *n* (%)	
T1	2 (1.9)
T2	18 (17.3)
T3	77 (74.0)
T4	7 (6.7)
Preoperative N stage, *n* (%)	
N0	43 (41.3)
N1	33 (31.7)
N2	18 (17.3)
NX	10 (9.6)
Preoperative M stage, *n* (%)	
M0	99 (95.2)
M1	5 (4.8)
Preoperative CRM involvement, *n* (%)	22 (21.2)

*BMI* Body mass index, *ASA* American Society of Anesthesiologists

### Surgical details

As shown in Table 2, 98 (94.2%) patients underwent taTME resection and primary anastomosis. Of these patients, 26.5% (26/98) and 92.9% (91/98) underwent manual anastomosis and defunctioning ileostomy, respectively. No patients underwent APR surgery. The remaining six patients (5.8%) underwent taTME resection and permanent colostomy. Two patients (1.9%, cases 31–32) were converted to laparotomy.

**Table 2:** Operation details and pathological and postoperative outcomes of all included patients

Variable	Values
**Operation details**	
Total operation time, min, median (IQR)	240 (210–290)
Intraoperative blood loss, mL, median (IQR)	100 (50–100)
Anastomotic technique, *n* (%)	
Stapled	68 (65.4)
Manual	26 (25.0)
Stapled and reinforced by manual	4 (3.8)
None	6 (5.8)
Stoma creation, *n* (%)	
None	7 (6.7)
Diverting loop ileostomy	91 (87.5)
Permanent colostomy	6 (5.8)
Transanal tube use, *n* (%)	72 (69.2)
Conversion to open, *n* (%)	2 (1.9)
**Pathology**	
Pathological T stage, *n* (%)	
(y)pT0	13 (12.5)
(y)pTis	3 (2.9)
(y)pT1	13 (12.5)
(y)pT2	25 (24.0)
(y)pT3	49 (47.1)
(y)pT4	1 (1.0)
Pathological N stage, *n* (%)	
(y)pN0	76 (73.1)
(y)pN1	20 (19.2)
(y)pN2	8 (7.7)
Tumor max size, mm, median (IQR)	30.0 (20.0–42.3)
Quality of TME specimen, *n* (%)	
Complete	78 (75.0)
Near complete	18 (17.3)
Incomplete	8 (7.7)
Circumferential margin involvement, *n* (%)	5 (4.8)
Distal margin involvement, *n* (%)	4 (3.8)
**Postoperative outcomes**	
Length of postoperative hospital stay, days, median (IQR)	7 (6–9)
Cases with complication(s) within 30 days, *n* (%)	39 (37.5)
Clavien–Dindo I, *n* (%)	7 (6.7)
Clavien–Dindo II, *n* (%)	25 (24.0)
Clavien–Dindo III, *n* (%)	4 (3.8)
Clavien–Dindo IV, *n* (%)	3 (2.9)
Clavien–Dindo V, *n* (%)	0
Anastomotic leakage, *n* (%)	17 (17.3)
Grade A	4 (4.1)
Grade B	12 (12.2)
Grade C	1 (1.0)
Reoperation, *n* (%)	3 (2.9)
Readmissions within 30 days, *n* (%)	8 (7.7)
Deaths within 30 days, *n* (%)	0

### Histopathological results

The histopathological results of the 104 patients are summarized in Table 2. Complete and near-complete TME specimens were obtained in 78 (75.0%) and 18 patients (17.3%), respectively, while incomplete TME specimens were reported in eight patients (7.7%). DRM- and CRM-positivity rates were 4.8% (5/104) and 3.8% (4/104), respectively. The median tumor size was 30.0 mm (*IQR*: 20.0–42.3).

### Postoperative complications

Postoperative outcome details are shown in the third module of Table 2. The median length of hospital stay was 7 days (*IQR*: 6–9). The incidences of postoperative and major complications (Clavien–Dindo ≥ III) within 30 days were 37.5% and 6.7%, respectively, and nine patients (8.3%) developed post-discharge complications. The incidence of AL was 17.3% (17/98), and three cases occurred after discharge. Of these patients, 70.6% (12/17) received interventional drainage or conservative treatment with antibiotics (grade B), and one patient underwent surgery (grade C). Overall, the 30-day readmission rate was 7.7% (8/108), including five AL-related cases. Three patients (2.9%) underwent reoperation, that is, cases 31, 62, and 63. The 31st case, due to intraoperative hemorrhage of the anterior sacral venous plexus, was treated for hemostasis with a cotton pad, which was also removed surgically. The remaining two patients underwent unplanned reoperations. A ureteral stent was implanted in the 62nd case because of urinary tract infection, and open debridement and drainage were performed in the 63rd case because of postoperative AL.

### Follow-up

The median follow-up period for the entire population was 28 months (*IQR*: 12–41.5). Local recurrence occurred in five patients (4.8%) during this period, of which one was DRM positive, one was CRM positive, and two died during follow-up. Nine patients (8.7%) developed distant metastasis, including two with simultaneous local recurrence and one with preoperative stage 4. The overall and cancer-related mortality rates were 7.7% (8/104) and 4.8% (5/104), respectively, with one death due to pulmonary infection 2 months after surgery, one death due to pulmonary embolism 2 months after surgery, and one death due to intestinal obstruction 15 months after surgery.

### CUSUM charts for the surgical outcomes

#### Surgical experience and operating time (Fig. 1)

**Fig. 1:**
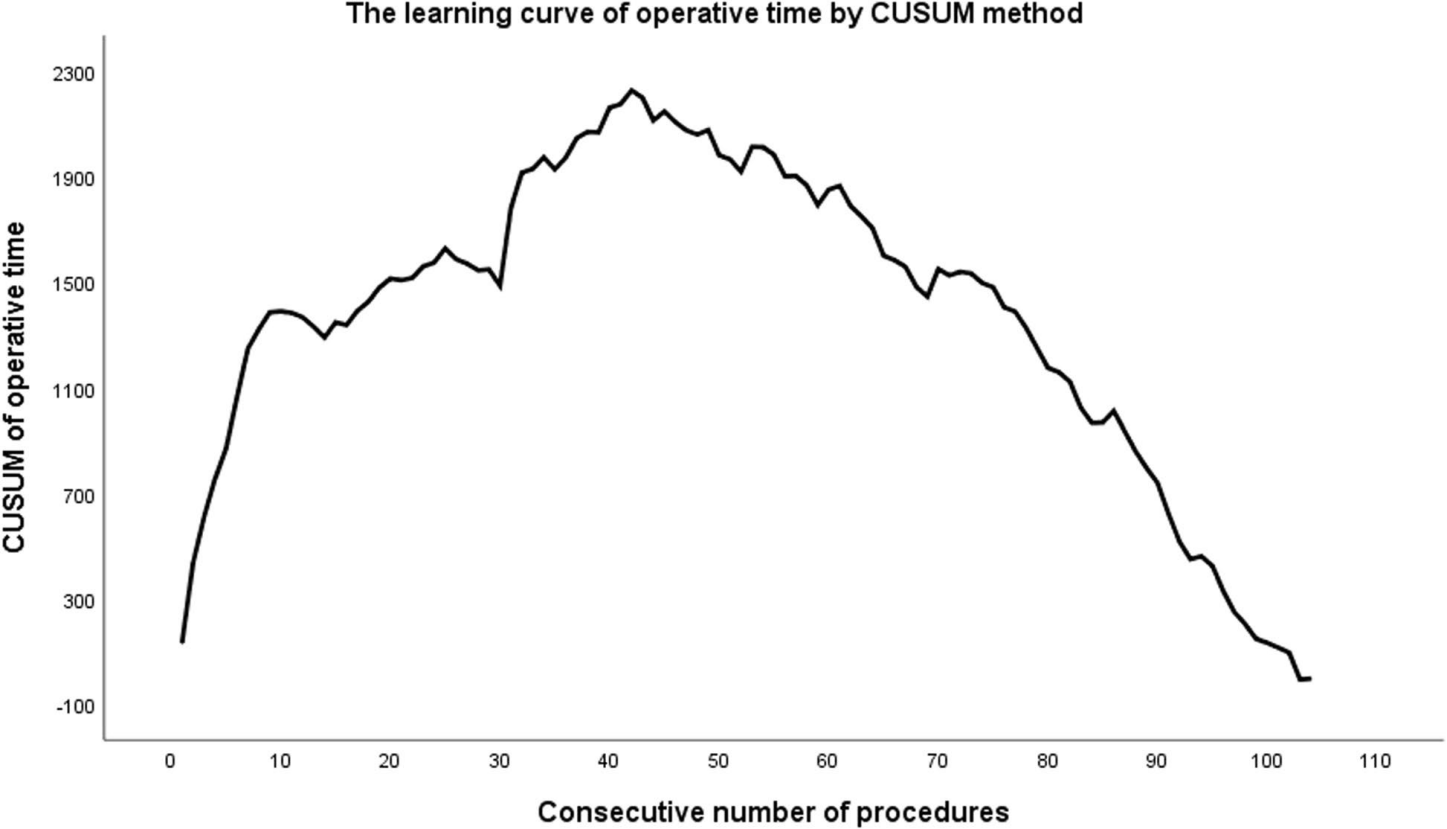
Cumulative sum curve for total operative time

The mean total operative time (transabdominal + transanal) in this study was 256.1 min (± 77.6). The CUSUM plot decreased when the operative time exceeded this value. According to the CUSUM operation-time diagram, the curve dropped in the initial stage in cases 10–14 and rose again after case 14, a phenomenon that may be related to the engagement of a new surgeon in the abdominal operation group. Based on a visual analysis of the learning curve, a peak was noted in the 42nd case (Fig. 1); therefore, case 42 was defined as the learning-curve cutoff point regarding surgical time, after which the learning curve declined. A comparison of operative time between the two phases (cases 1–42 and cases 43–104) confirmed significant differences and a gradual decrease in operative time after case 42 (309.1 ± 84.4 vs 220.2 ± 46.4, *P* <0.001) (Table 4). In the initial stage, manual anastomosis accounted for a high proportion of patients (35.7% vs. 17.7%, *P* = 0.038) (Table 4). Sex, BMI, complications, ASA grade, history of abdominal and pelvic surgery, tumor location, and neoadjuvant therapy exhibited no statistical differences between the two stages (Table 3). There were no significant differences in intraoperative blood loss, conversion rate to open surgery, integrity of mesangial specimen, and CRM and DRM statuses between the two stages (Table 4).

**Table 3:** Patient demographics, preoperative staging, and treatment according to the inflection points in the three learning curves

Variable	Operation-time group		***P***	Postoperative-complication group			Anastomotic-leakage group		***P***
Consecutive no. of patients	1–42	43–104		1–43	44–75	76–104	1–68	69–98	
Sex, *n* (%)			0.186						0.395
Male	34 (81.0%)	43 (69.4%)		35 (81.4%)	23 (71.9%)	19 (65.5%)	51 (75.0%)	20 (66.7%)	
Female	8 (19.0%)	19 (30.6%)		8 (18.6%)	9 (28.1%)	10 (34.5%)	17 (25.0%)	10 (33.3%)	
Age, mean (SD)	64.5 (10.7)	59.5 (11.7)	0.028	63.8 (11.4)	58.6 (10.8)	61.2 (12.0)	60.8 (10.5)	60.4 (12.6)	0.883
BMI, kg/m^2^, median (IQR)	24.2 (21.6–26.1)	24.1 (22.5–26.0)	0.691	24.2 (21.6–26.1)	24.6 (22.6–26.2)	23.9 (22.2–26.0)	24.4 (22.4–26.1)	23.9 (22.3–26.0)	0.988
Comorbidity, *n* (%)	22 (52.4%)	26 (41.9%)	0.294	22 (51.2%)	12 (37.5%)	14 (48.3%)	29 (42.6%)	14 (46.7%)	0.712
ASA, *n* (%)			0.058						0.745
I	6 (14.3%)	3 (4.8%)		6 (14.0%)	1 (3.1%)	2 (6.9%)	6 (8.8%)	3 (10.0%)	
II	26 (61.9%)	51 (82.3%)		27 (62.8%)	26 (81.2%)	24 (82.8%)	51 (75.0%)	24 (80.0%)	
III	10 (23.8%)	8 (12.9%)		10 (23.3%)	5 (15.6%)	3 (10.3%)	11 (16.2%)	3 (10.0%)	
Neoadjuvant therapy, *n* (%)	18 (42.9%)	33 (53.2%)	0.299	19 (44.2%)	23 (71.9%)	9 (31.0%)&	40 (58.8%)	9 (30.0%)	0.009
Previous abdominal surgery, *n* (%)	10 (23.8%)	11 (17.7%)	0.449	10 (23.3%)	7 (21.9%)	4 (13.8%)	15 (22.1%)	4 (13.3%)	0.314
Distance from anal verge, mm, median (IQR)	48.0 (40.2–58.0)	52.5 (39.8–70.8)	0.428	47.0 (40.0–58.0)	46.0 (38.8–64.0)	58.0 (43.0–77.0)	46.5 (40.0–58.5)	59.0 (43.0–79.2)	0.035
Middle rectum, *n* (%)	16 (38.1%)	32 (51.6%)	0.175	16 (37.2%)	13 (40.6%)	19 (65.5%)	26 (38.2%)	20 (66.7%)	0.009
Lower rectum, *n* (%)	26 (61.9%)	30 (48.4%)	0.175	27 (62.8%)	19 (59.4%)	10 (34.5%)	42 (61.8%)	10 (33.3%)	0.009
Preoperative CRM involvement, *n* (%)	10 (23.8%)	12 (19.4%)	0.585	11 (25.6%)	8 (25.0%)	3 (10.3%)	18 (26.5%)	3 (10.0%)	0.067

*P < 0.05, comparison between phases 2 and 1. #*P* < 0.05, comparison between phases 3 and 1. &*P* < 0.05, comparison between phases 3 and 2

**Table 4:** Surgical and pathological characteristics according to the inflection points in the three learning curves

Variable	Operation-time group		***P***	Postoperative-complication group			Anastomotic-leakage group		***P***
Consecutive. of patients	1–42	43–104		1–43	44–75	76–104	1–68	69–98	
Total operation time, min, median (IQR)	295.0 (251.0–330.0)	220.0 (180.0–240.0)	< 0.001	290.0 (250.–330.0)	228.5 (211.5–255.5)	199.0 (180.0–238.0)	256.0 (220.0–308.5)	199.5 (180.0–239.5)	< 0.001
Intraoperative blood loss, mL, median (IQR)	100.0 (57.5–200.0)	100.0 (50.0–100.0)	0.173	100.0 (65.0–200.0)	100.0 (50.0–100.0)	100.0 (50.0–100.0)	100.0 (50.0–100.0)	100.0 (50.0–100.0)	0.491
Anastomotic technique, *n* (%)			< 0.001						0.092
Manual	15 (35.7%)	11 (17.7%)		16 (37.2%)	6 (18.8%)	4 (13.8%)	22 (32.4%)	4 (13.3%)	
Stapled	18 (42.9%)	50 (80.6%)		18 (41.9%)	26 (81.2%)^*^	24 (82.8%)#	43 (63.2%)	25 (83.3%)	
Stapled and reinforced manually	3 (7.1%)	1 (1.6%)		3 (7.0%)	0 (0.0%)	1 (3.4%)	3 (4.4%)	1 (3.3%)	
None	6 (14.3%)	0 (0.0%)		6 (14.0%)	0 (0.0%)	0 (0.0%)	NA	NA	
Stoma creation, *n* (%)			0.003						0.010
None	1 (2.4%)	5 (8.1%)		1 (2.3%)	0 (0.0%)	5 (17.2%)&	29 (42.6%)	0 (0.0%)	
Diverting loop ileostomy	35 (83.3%)	57 (91.9%)		36 (83.7%)	32 (100.0%)*	24 (82.8%)&	39 (57.4%)	30 (100.0%)	
Permanent colostomy	6 (14.3%)	0 (0.0%)		6 (14.0%)	0 (0.0%)	0 (0.0%)	NA	NA	
Transanal tube use, *n* (%)	18 (42.9%)	54 (87.1%)	< 0.001	19 (44.2%)	24 (75.0%)^*^	29 (100.0%)#&	39 (57.4%)	30 (100.0%)	< 0.001
Conversion to open, *n* (%)	2 (4.8%)	0 (0.0%)	0.161	2 (4.7%)	0 (0.0%)	0 (0.0%)	0	0	NA
Reoperation, *n* (%)	1 (2.4%)	2 (3.2%)	0.801	1 (2.3%)	2 (6.2%)	0 (0.0%)	2 (2.9%)	0 (0.0%)	1.0

*P < 0.05, comparison between phases 2 and 1. #*P* < 0.05, comparison between phases 3 and 1. &*P* < 0.05, comparison between phases 3 and 2

#### Surgical experience and postoperative complications (Fig. 2)

**Fig. 2:**
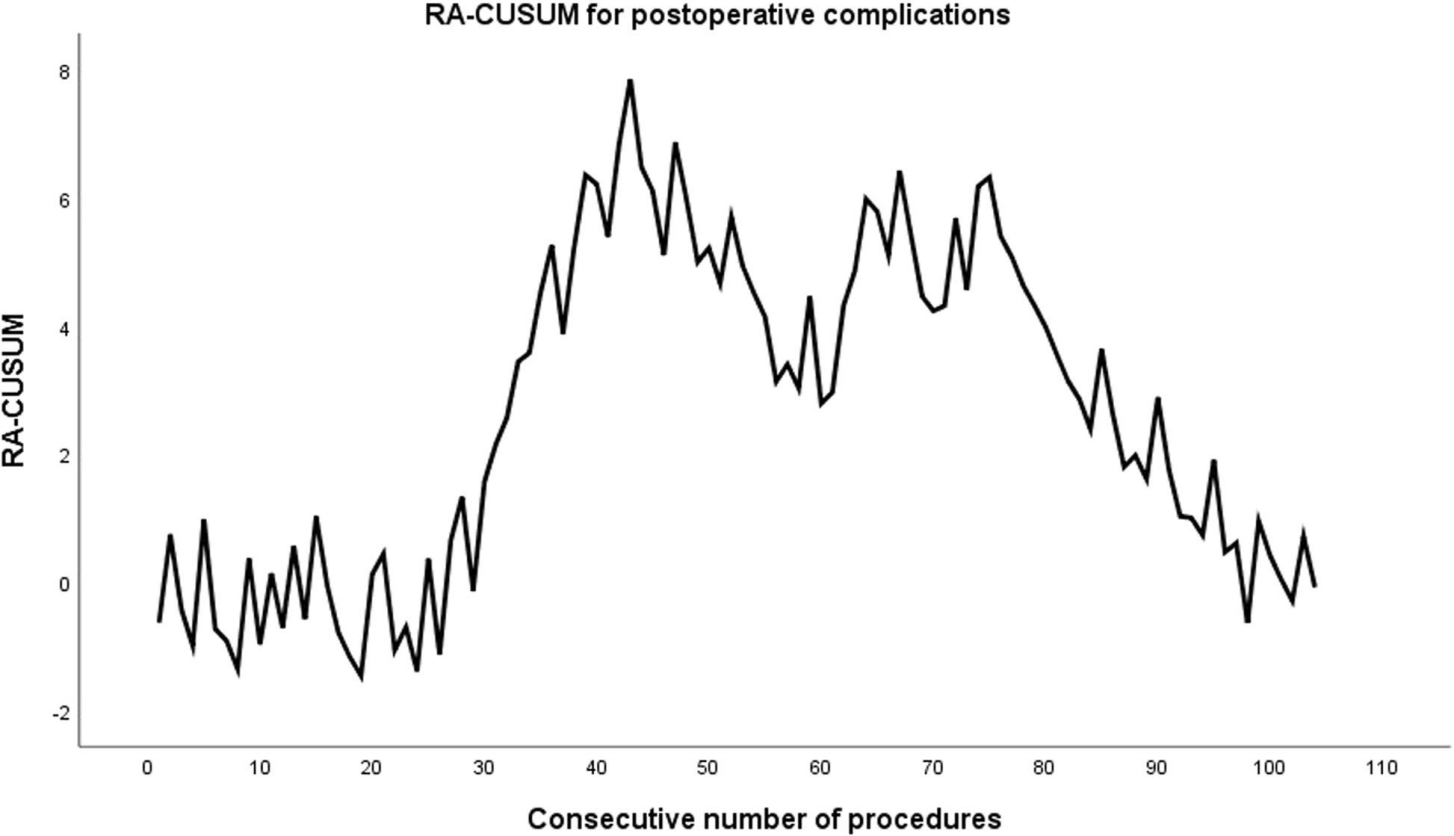
Risk-adjusted cumulative sum (RA-CUSUM) curve for postoperative complications

Postoperative complications occurred within 30 days in 39 patients (37.5 %). The RA-CUSUM for postoperative complications revealed changes in cases 43 and 75 (Fig. 2). As shown in the RA-CUSCUM diagram, the curve realized a significant upward trend from case 1 to 43 and the highest peak at case 43. The curve fluctuated significantly from the 44th to the 75th case, and after the 75th case, the curve generally showed a downward trend. Therefore, the learning curve could be divided into three phases based on the endpoint of postoperative complications: phase 1 (case 1–43), phase 2 (cases 44–75), and phase 3 (cases 76–104). See Tables 3, 4 and 5 for detailed information regarding comparisons of the three phases.

**Table 5:** Postoperative outcomes according to the inflection points in the three learning curves

Variable	Operation-time group		***P***	Postoperative-complication group			Anastomotic-leakage group		***P***
Consecutive patients	1–42	43–104		1–43	44–75	76–104	1–68	69–98	
Hospital stay, days, median (IQR)	7.0 (6.0–12.0)	7.0 (6.0–8.0)	0.217	7.0 (6.0–12.0)	7.0 (6.0–8.2)	7.0 (6.0–8.0)	7.0 (6.0–9.0)	7.0 (6.0–8.0)	0.636
Cases with complication(s) within 30 days, *n* (%)	22 (52.4%)	17 (27.4%)	0.010	23 (53.5%)	11 (34.4%)	5 (17.2%)&	28 (41.2%)	6 (20.0%)	0.042
Clavien–Dindo I-II, *n* (%)	20 (47.6%)	12 (19.4%)	0.009	21 (48.8%)	8 (25.0%)	3 (10.3%)#	25 (36.8%)	3 (10.0%)	0.021
Clavien–Dindo III-IV, *n* (%)	2 (4.8%)	5 (8.1%)	0.009	2 (4.7%)	3 (9.4%)	2 (6.9%)	3 (4.4%)	3 (10.0%)	0.011
Anastomotic leakage, *n* (%)	8 (19.0%)	9 (14.5%)	0.540	9 (20.9%)	6 (18.8%)	2 (6.9%)	15 (22.1%)	2 (6.7%)	0.064
Reoperation, *n* (%)	1 (2.4%)	2 (3.2%)	1.0	1 (2.3%)	2 (6.2%)	0 (0.0%)	2 (2.9%)	0 (0.0%)	1.00
Readmissions within 30 days, *n* (%)	6 (14.3%)	2 (3.2%)	0.059	6 (14.0%)	1 (3.1%)	1 (3.4%)	6 (8.8%)	2 (6.7%)	1.00

*P < 0.05, comparison between phases 2 and 1. #*P* < 0.05, comparison between phases 3 and 1. &*P* < 0.05, comparison between phases

The incidences of complications in the three phases were 53.5%, 34.4%, and 17.2%, respectively (Table 5). The incidence of postoperative complications was significantly lower in the third phase than in the first phase, and it was lower in the second phase than in the first phase; however, the differences were not statistically different. The three phases were similar in terms of major postoperative complications. The rates of neoadjuvant therapy in the three phases were 44.2%, 71.9%, and 31.0%, respectively, and there was a statistically significant difference between the third and second phases (Table 3). The rates of stapled anastomosis in the three phases were 41.9%, 81.3%, and 82.8%, respectively, and those in the second and third phases were significantly higher than those in the first phase (Table 5). The rate of rectal tube use increased gradually in the three phases, and there were statistically significant differences between the three phases (44.2% vs. 75.0% vs. 100.0%) (Table 4). No statistically significant differences were observed in the patients’ demographic data, tumor size, location, and stage (Table 3). Other short-term outcomes, including conversion to open surgery, reoperation, and readmission, did not differ significantly among the three phases.

#### Surgical experience and AL (Fig. 3)

The incidence of AL within 30 days of surgery was 17.3%. With operation time as the study outcome, the AL rate in the first 42 cases was 19.0%, and that in the latter 42 cases was 14.5%, revealing no statistically significant difference (*P* = 0.540; Table 5). With postoperative complications as the study outcome, AL rates in the three phases were 20.9%, 18.8%, and 6.9%, respectively (Table 5). In conclusion, although there was no statistically significant difference, the incidence of AL gradually decreased with an increase in the number of surgical cases.

**Fig. 3:**
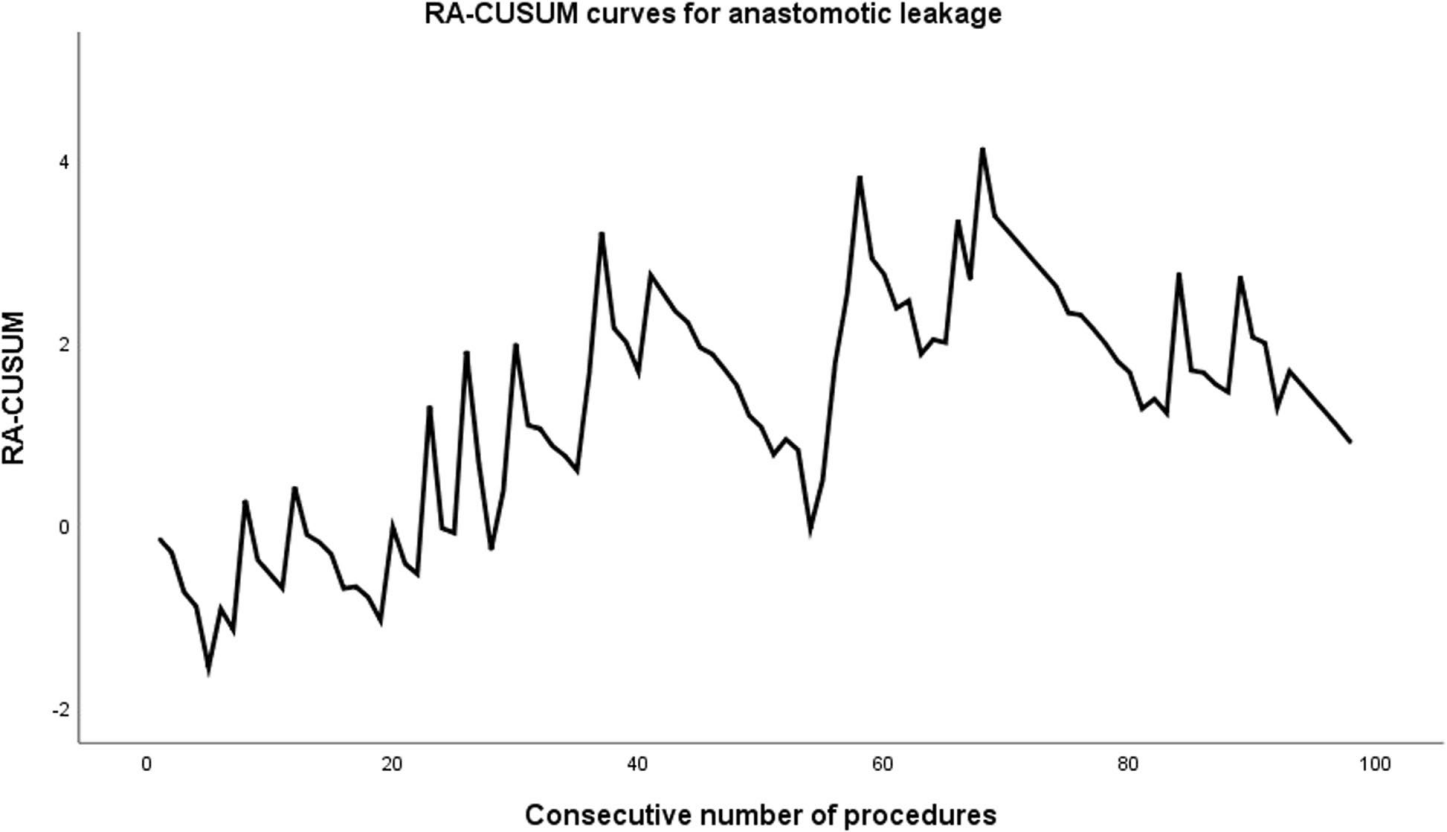
Risk-adjusted cumulative sum (RA-CUSUM) curve for anastomotic leakage

In the RA-CUSUM plot, among the 98 patients undergoing anastomosis, the curve for cases 1–68 demonstrated an upward trend and peaked at the 68th case. Although there were obvious fluctuations during this period, the curve presented a downward trend after case 68. Therefore, the 68th case was used as the cutoff for the curve, and the AL rates in the two phases (1–68 and 69–98) were 22.1% and 6.7%, respectively (*P* = 0.064; Table 5).

As shown in Tables 3 and 4, the distance of the lower tumor margin from the anal margin (50.7 ± 15.5 vs 60.6 ± 23.1%, *P* = 0.014), neoadjuvant therapy (58.8% vs. 30.0%, *P* = 0.009), instrument anastomosis (63.2% vs. 83.3%, *P* = 0.047), and anal drainage (57.4% vs. 100.0%, *P* < 0.001) were statistically different between the two phases. There were no significant differences in sex, age, BMI, ASA grade, or tumor T stage.

## Discussion

The analysis was based on a consecutive series of 104 mid-low rectal cancer patients who underwent taTME. The evaluation of total operation time revealed a clear learning curve, which improved significantly after 42 cases. The results showed a learning curve for postoperative complications consisting of two peaks, which could be considered three predictable turning points. The first peak, at the 43rd procedure, is a “fluctuating” trend in cases 44–75. The second peak, at the 75th procedure, could be considered the point at which “mastery” is reached. Learning curve analysis was performed after six cases without anastomosis were excluded, and the curve shows a significant downward trend after the 68th case.

Among the published studies on the taTME learning curve, two studies adopted operation time as the study outcome for analysis. Koedam et al. [17] studied the learning curve of postoperative outcomes in 138 patients undergoing different surgical approaches, including one- and two-team approaches, and they defined operation time as the time from incision to completion of wound closure. The CUSUM curve exhibited a downward trend in operation time around the 80th case, which was associated with a shift to the two-team approach. Persiani et al. [11] conducted a learning-curve analysis based on a consecutive cohort of 121 patients with mid-low rectal cancer who underwent taTME by the same surgical team. The first eight cases underwent surgery using a two-team approach, whereas, in the ninth case, a synchronous two-team approach was applied. The study noted that “mastery” of the procedure was achieved on the 71st occasion. However, the switch between the one-team and two-team approaches is bound to have a potential impact on learning-curve analysis. In addition, in the two-team approach, when surgery was performed simultaneously or sequentially in the transabdominal and transanal groups, the timing of the intervention in the transanal group affected the operation time. Therefore, in this study, the sum of the operation times in the transanal and transabdominal groups was used for CUSUM analysis to determine the learning curve.

In this study, based on the analysis of operation time as the outcome, the CUSUM curve graph exhibited a downward trend after the completion of 42 cases, and the operation time decreased significantly after this changeover compared with before. There were no significant differences in baseline characteristics between the two phases (1–42 and 43–104). Therefore, a surgeon can be considered to have achieved “proficiency” in the taTME procedure after completing 42 cases.

In the early phases of the procedure, various surgical platforms, operating equipment, and inflatable devices are used to determine the appropriate surgical mode. In addition, as shown in Fig. 1, the curve rose again at the 14th case when the transabdominal surgeon changed, an occurrence that might have had a certain impact on the learning curve. In view of this, it can be speculated that, for beginners, using sophisticated surgical equipment and a fixed assistant team is potentially helpful in shortening the learning curve.

Operation time is often used to study learning curves; nonetheless, a shorter operation time does not necessarily result in improved clinical outcomes [18]. An improvement in the clinical outcomes of patients reflects an improvement in surgical techniques. Therefore, in addition to surgical time, postoperative complications and AL were included in the present study for learning-curve analysis.

The incidence of postoperative complications was 37.5%. Learning-curve analysis using RA-CUSUM demonstrated that postoperative complications in the first 43 patients had the largest increase, and an obvious fluctuation between the 44th and 75th patients occurred. After the 75th patient, the curve generally showed a downward trend. Although the incidence of postoperative complications decreased in the second phase (44–75) compared with that in the first phase (1–43), the difference was not statistically significant. The incidence of postoperative complications was significantly lower in cases 43–104 than in cases 1–42 (53.3% vs. 24.4%, *P* = 0.007) according to intergroup comparisons based on operative time. However, the RA-CUSUM learning curve for postoperative complications showed a “fluctuating” trend in cases 44–75. Surgeons have been reported to select more challenging cases as they become more experienced [11]. Patients receiving neoadjuvant therapy often have more complex tumor conditions. After chemoradiotherapy, it is often difficult to obtain the correct resection plane visual field during surgery because of the poor visibility of the anatomical plane [19, 20]. Some studies have suggested that neoadjuvant therapy may be associated with an increase in postoperative rectal-cancer complications [21, 22]. In this study, the rate of neoadjuvant therapy increased at this phase (44–75) compared with the first stage (71.9% vs 44.2%), possibly justifying the nonsignificant decrease in postoperative complications and “fluctuation” in the learning curve in phase 44–75. In light of this, once a surgeon achieves “proficiency” in the procedure, it may entail overcoming certain challenges before “mastery” of the whole procedure is achieved. Beginners are encouraged to avoid selecting “difficult cases” before completely crossing the learning curve.

The risk of post-AL permanent stoma was > 60% and significantly associated with poor overall survival, disease-free survival, and cancer-specific survival [23]. Therefore, it is equally important to explore the influence of the learning curve on post-taTME AL occurrence. In this study, the incidence of AL at 30 days was 17.3%. RA-CUSUM analysis was performed after six cases without anastomosis were excluded, and the curve peaked at the 68th case. The AL rates in the two phases (1–68 and 69–98) were 22.1% and 6.7%, respectively (*P* = 0.064). The AL rate decreased significantly in the second phase (69–98), although there was no statistical difference, a phenomenon that may be related to the small number of cases in the second phase. As the procedure progressed, the rate of rectal tube use (57.4% vs. 100.0%, *P* < 0.001) continued to rise, thus possibly contributing to AL reduction [24]. A high proportion of manual anastomosis in the early stages potentially influences AL occurrence [16]. For beginners, these experiences may be helpful in reducing AL. In addition, Fig. 3 shows that the curve declined significantly after reaching the first peak in the 37th case but continued to exhibit a “fluctuation” trend in the case 37–68 phase, reaching its peak in the 68th case. This trend is relatively similar to the RA-CUSUM curve for postoperative complications.

Conversion to open surgery, reoperation, readmission, and major postoperative (surgical) complications (Clavien–Dindo ≥ III) may be preferred learning-curve outcomes, as they verify the safety of the procedure and are of critical importance from a patient’s perspective. In this study, the conversion rates to open surgery, reoperation, and readmission were 1.9%, 2.9%, and 7.7%, respectively, figures that are comparable to those reported in international large-sample studies [25, 26]. Owing to the small sample size, potential learning curves could be analyzed based on these data. The incidence of major postoperative complications was 6.7%, two of which were nonsurgically related, and it was considerably lower than the 28.2% rate of severe complications reported by Koedam et al. [17] in the taTME learning curve. Therefore, this study did not analyze the learning curve of postoperative severe (surgical) complications as a study outcome.

Poor-quality resection was performed in 10.6% (11/104) of cases, including positive CRM in 4.8% (5/104), positive DRM 3.8% (4/104), and incomplete TME in 7.7% (8/104). The specimen quality results of this study were comparable to the current reports [27, 28]. Lee et al. [29] used a composite outcome of margins’ status and completeness of the specimen to determine the learning curve of taTME, and this study showed that taTME requires a minimum of 45–51 cases to reach an acceptable incidence of high-quality TME and lower operative duration. The results were consistent with the number of cases required to achieve proficiency in this study. A risk-adjusted CUSUM analysis with specimen quality as outcome was also conducted (Fig. S2). The curve trend was relatively similar to the RA-CUSUM curve for postoperative complications. However, due to the low incidence, no risk factors were found in univariate and multivariate analysis, which may reduce the reliability of results. Therefore, specimen quality was not included in the outcomes.

The main advantage of this study is that, as our center is one of the earliest centers to conduct taTME procedures in China, it has experienced several challenges and attempts in the early stages, and this experience is potentially useful as a reference to beginners preparing to perform taTME procedures. Furthermore, the study used the sum of transabdominal and transanal operation time as the total operation time so as to avoid the influence of operation-approach switching and surgical-team connection on the operation time.

Notwithstanding, this study has certain limitations. Considering that our center is one of the first to perform this operation in China, colorectal surgeons in China had not yet established a mature plan for reference in the early stages of development, and the surgical team made many attempts, such as surgical platforms, inflatable devices, and operating instruments, which might have had a potential impact on the learning-curve findings. Although the study included a reasonable number of cases, it was limited by the small number of events related to major (surgical) complications (Clavien–Dindo ≥ III), reoperation, and conversion to open surgery, among others, in conducting a learning-curve analysis. Finally, since follow-up was limited, and only short-term outcomes were included in the analysis, further studies are required to analyze long-term outcomes as well as the learning curves of other surgeons to validate the existing conclusions.

## Conclusions

The evaluation of total operation time revealed a clear learning curve, which improved significantly after 42 cases. However, the learning curves for postoperative complications and AL were optimized after 75 and 68 surgeries, respectively. According to our findings, after surgeons become “proficient” in the operation process, they occasionally encounter some “challenging” cases; however, this often causes postoperative complications. Furthermore, surgeons may require ≥ 75 cases to achieve “mastery” in the taTME procedure.

## Supplementary Information

**Additional file 1: Figure s1a.** Star-port platform specific operation. Fig. s1b. Star-port platform before assembling. Fig. s1c. Star-port platform after assembling. **Figure s2.** Risk-adjusted cumulative sum (RA-CUSUM) curve for specimen quality.

## Data Availability

Datasets are available on request from the corresponding author on reasonable request. The raw data and all related documents supporting the conclusions of this manuscript will be made available by the authors, without undue reservation, to any qualified researcher.

## References

[1] Sylla P, Rattner DW, Delgado S, Lacy AM (2010). NOTES transanal rectal cancer resection using transanal endoscopic microsurgery and laparoscopic assistance. Surg Endosc..

[2] Heald RJ (2013). A new solution to some old problems: transanal TME. Tech Coloproctol..

[3] Aallah S, Albert M, Larach S (2010). Transanal minimally invasive surgery: a giant leap forward. Surg Endosc..

[4] Adamina M, Buchs NC, Penna M, Hompes R, St. (2018). Gallen Colorectal Consensus Expert Group. St. Gallen consensus on safe implementation of transanal total mesorectal excision. Surg Endosc..

[5] Wasmuth HH, Faerden AE, Myklebust TÅ, Pfeffer F, Norderval S, Riis R (2020). Transanal total mesorectal excision for rectal cancer has been suspended in Norway. Br J Surg..

[6] Van Oostendorp SE, Belgers HJ, Bootsma BT, Hol JC, Belt EJTH, Bleeker W (2020). Locoregional recurrences after transanal total mesorectal excision of rectal cancer during implementation. Br J Surg..

[7] Caycedo-Marulanda A, Lee L, Chadi SA, Verschoor CP, Crosina J, Ashamalla S (2021). Association of transanal total mesorectal excision with local recurrence of rectal cancer. JAMA Netw Open..

[8] Lau S, Kong J, Bell S, Heriot A, Stevenson A, Moloney J (2021). Transanal mesorectal excision: early outcomes in Australia and New Zealand. Br J Surg..

[9] Bege T, Lelong B, Esterni B, Turrini O, Guiramand J, Francon D (2010). The learning curve for the laparoscopic approach to conservative mesorectal excision for rectal cancer: lessons drawn from a single institution’s experience. Ann Surg..

[10] Nagtegaal ID, Van De Velde CJ, Van Der Worp E, Kapiteijn E, Quirke P, van Krieken JH (2002). Macroscopic evaluation of rectal cancer resection specimen: clinical significance of the pathologist in quality control. J Clin Oncol..

[11] Persiani R, Agnes A, Belia F, D’Ugo D, Biondi A (2021). The learning curve of TaTME for mid-low rectal cancer: a comprehensive analysis from a five-year institutional experience. Surg Endosc..

[12] Clavien PA, Barkun J, De Oliveira ML, Vauthey JN, Dindo D, Schulick RD (2009). The Clavien-Dindo classification of surgical complications: five-year experience. Ann Surg..

[13] Rahbari NN, Weitz J, Hohenberger W, Heald RJ, Moran B, Ulrich A (2010). Definition and grading of anastomotic leakage following anterior resection of the rectum: a proposal by the International Study Group of Rectal Cancer. Surgery..

[14] Bolsin S, Colson M (2000). The use of the Cusum technique in the assessment of trainee competence in new procedures. Int J Qual Health Care..

[15] Steiner SH, Cook RJ, Farewell VT, Treasure T (2000). Monitoring surgical performance using risk-adjusted cumulative sum charts. Biostatistics..

[16] Penna M, Hompes R, Arnold S, Wynn G, Austin R, Warusavitarne J (2019). Incidence and risk factors for anastomotic failure in 1594 patients treated by transanal total mesorectal excision: results from the international TaTME registry. Ann Surg..

[17] Koedam TWA, Veltcamp Helbach M, Van De Ven PM, Kruyt PM, van Heek NT, Bonjer HJ (2018). Transanal total mesorectal excision for rectal cancer: evaluation of the learning curve. Tech Coloproctol..

[18] Chen W, Sailhamer E, Berger DL, Rattner DW (2007). Operative time is a poor surrogate for the learning curve in laparoscopic colorectal surgery. Surg Endosc..

[19] Bonjer HJ, Deijen CL, Abis GA, Cuesta MA, van der Pas MH, de Lange-de Klerk ES (2015). A randomized trial of laparoscopic versus open surgery for rectal cancer. N Engl J Med..

[20] Alhanafy MK, Park SS, Park SC, Park B, Kim MJ, Sohn DK (2020). Early experience with transanal total mesorectal excision compared with laparoscopic total mesorectal excision for rectal cancer: a propensity score-matched analysis. Dis Colon Rectum..

[21] Berkel AE, Woutersen DP, Van Der Palen J, Klaase JM (2014). Prognostic factors for postoperative morbidity and tumour response after neoadjuvant chemoradiation followed by resection for rectal cancer. J Gastrointest Surg..

[22] Fleming FJ, Påhlman L, Monson JR (2011). Neoadjuvant therapy in rectal cancer. Dis Colon Rectum..

[23] Bashir Mohamed K, Hansen CH, Krarup PM, Fransgård T, Madsen MT, Gögenur I (2020). The impact of anastomotic leakage on recurrence and long-term survival in patients with colonic cancer: a systematic review and meta-analysis. Eur J Surg Oncol..

[24] Choy KT, Yang TWW, Heriot A, Warrier SK, Kong JC (2021). Does rectal tube/transanal stent placement after an anterior resection for rectal cancer reduce anastomotic leak? A systematic review and meta-analysis. Int J Colorectal Dis..

[25] Penna M, Hompes R, Arnold S, Wynn G, Austin R, Warusavitarne J (2017). Transanal total mesorectal excision: international registry results of the first 720 cases. Ann Surg..

[26] Ose I, Perdawood SK (2021). A nationwide comparison of short-term outcomes after transanal, open, laparoscopic, and robot-assisted total mesorectal excision. Colorectal Dis..

[27] de Lacy FB, van Laarhoven JJEM, Pena R, Arroyave MC, Bravo R, Cuatrecasas M, Lacy AM (2018). Transanal total mesorectal excision: pathological results of 186 patients with mid and low rectal cancer. Surg Endosc..

[28] Perdawood SK, Kroeigaard J, Eriksen M, Mortensen P (2021). Transanal total mesorectal excision: the Slagelse experience 2013-2019. Surg Endosc..

[29] Lee L, Kelly J, Nassif GJ, deBeche-Adams TC, Albert MR, Monson JRT (2020). Defining the learning curve for transanal total mesorectal excision for rectal adenocarcinoma. Surg Endosc..

