# Supplementary material for: The taTME learning curve for mid-low rectal cancer: a single-center experience in China
Source: World J Surg Oncol. 2022 Sep 23;20:305. doi: 10.1186/s12957-022-02763-3 (PMC9502600; doi:10.1186/s12957-022-02763-3)
Supplement: Supplementary file 1 — Additional file 1: Figure s1a. Star-port platform specific operation. Fig. s1b. Star-port platform before assembling. Fig. s1c. Star-port platform after assembling. Figure s2. Risk-adjusted cumulative sum (RA-CUSUM) curve for specimen quality. [file 12957_2022_2763_MOESM1_ESM.docx]

**Supplementary Information**

**The taTME learning curve for mid-low rectal cancer: a single-center experience in China**

Xu Feng-ming^1#^, Zhang Yi-qiao^1#^, Yan Jia-fu^2^, Xu Bo-wen^1^, Wu Guo-cong^1^, Yang Zheng-yang^1^, Sun Li-ting1, Zhang Xiao1, Yao Hong-wei1, Zhang Zhong-tao1

**Affiliations:**

Xu Feng-ming, Zhang Yi-qiao contributed equally to this article

^1^ Department of General Surgery, Beijing Friendship Hospital, Capital Medical University, Beijing 100050, China

^2^ Department of Cardiovascular Medicine, Beijing Anzhen Hospital, Capital Medical University, Beijing 100029, China

**Supplementary Figures：**


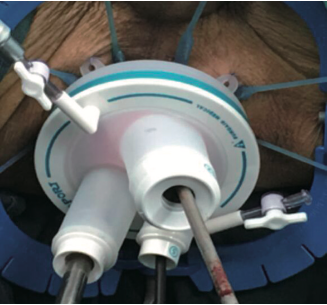


**Fig. s1a.** Star-port platform specific operation


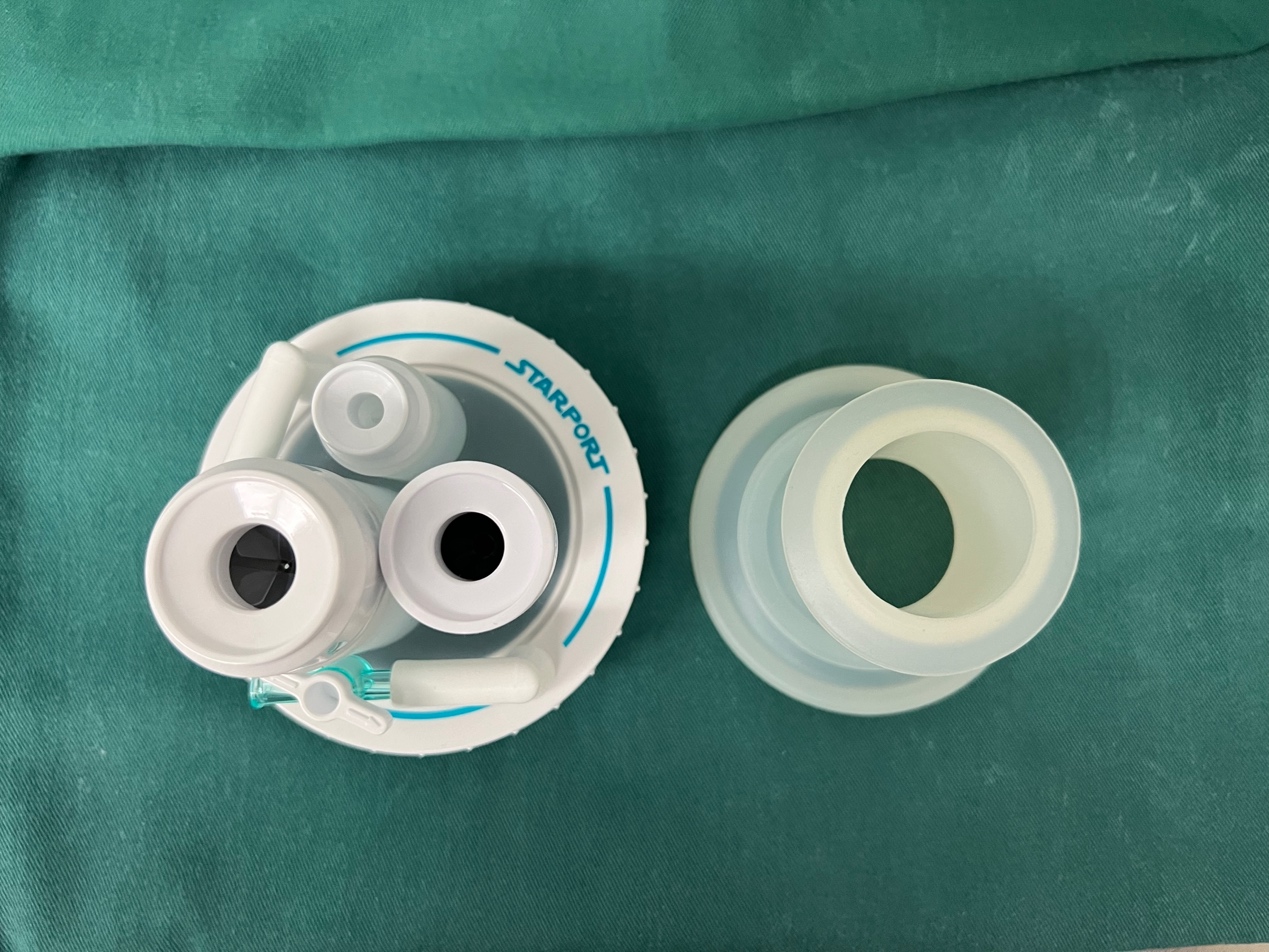


**Fig. s1b.** Star-port platform before assembling


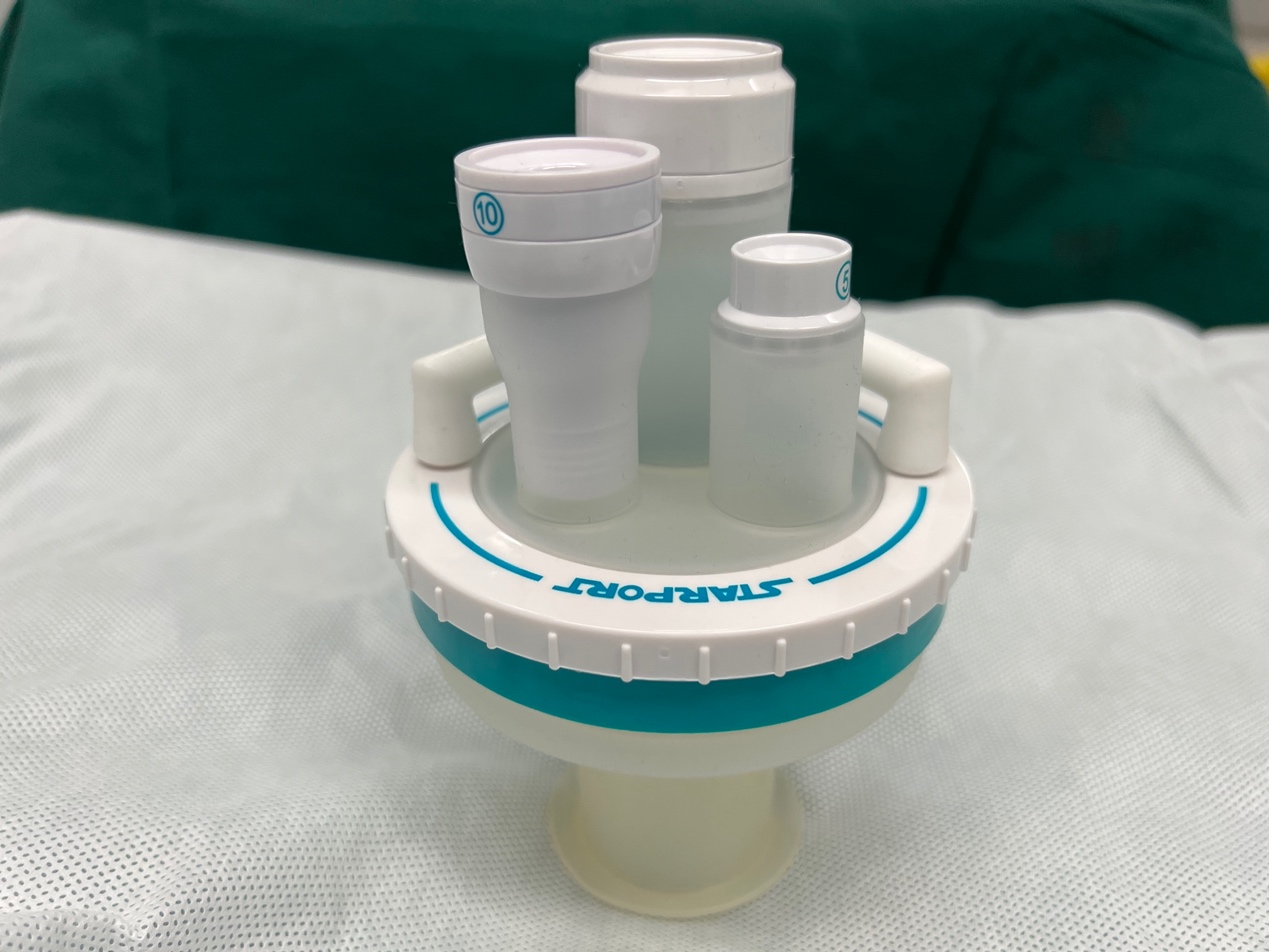


**Fig. s1c.** Star-port platform after assembling


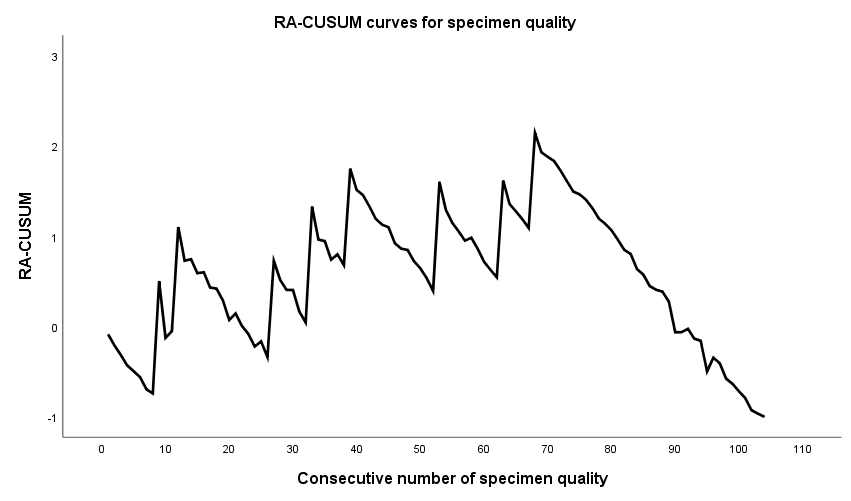


**Fig. s2.** Risk-adjusted cumulative sum (RA-CUSUM) curve for specimen quality
